# Personal Health Record (PHR) Experience and Recommendations for a Transformation in Saudi Arabia

**DOI:** 10.3390/jpm13081275

**Published:** 2023-08-19

**Authors:** Abdullah Alanazi, Mohammed Alanazi, Bakheet Aldosari

**Affiliations:** 1 Health Informatics Department, King Saud Ibn Abdulaziz University for Health Sciences, P.O. Box 3660, Riyadh 11481, Saudi Arabiadosarib@gmail.com (B.A.); 2 King Abdullah International Medical Research Center, P.O. Box 3660, Riyadh 11481, Saudi Arabia

**Keywords:** Personal Health Record (PHR), adoption, quality of care, transform

## Abstract

A Personal Health Record (PHR) is a patient-managed platform for health data. Most hospitals provide a PHR as an extension of the Electronic Medical Record (EMR). However, there are unresolved issues around the adoption rate, functionalities, barriers and, more importantly, the impact of the PHR on patients’ health. A cross-sectional, survey-based descriptive study was conducted in which patients from four main tertiary hospitals in Saudi Arabia were targeted from September 2022 to February 2023. The survey was tested and validated to address the objectives of the study. The survey covered components related to intention to use the PHR, required functions, obstacles and expected outcomes. This study involved 396 participants from the top four hospitals. It was discovered that the majority of them had intentions to use the PHR (93%) and believed it to be useful (94%) and easy to use (94%). It was widely agreed that accessing medical records (77%), scheduling appointments (88%), renewing medication (90%), tracking patient data (70%) and receiving personalized education (78%) were essential aspects of the PHR. Furthermore, the survey revealed that 54% of respondents saw a positive effect on their health status. A significant number of participants, around 54%, expressed concerns about the privacy of their PHR, and 46% reported concerns about the accuracy of their information. The study found that demographic factors and the type of hospital did not have a statistically significant association with the intention to use the PHR. Our findings showed that there were no significant barriers to adopting the PHR. Additionally, we found that less than half of the participants believed that their current PHR helped them to improve their health. This highlights the need for healthcare organizations to focus on improving the PHR’s functionality and overall purpose. Instead of simply providing basic features, the PHR should allow patients to manage their health information comprehensively, including compiling information from hospitals and patient-generated data. Having a PHR is crucial in improving an individual’s overall health. As technology advances, more data are being generated that should be included in the PHR to ensure an accurate and comprehensive view of the patient’s health. Expanding the scope of the PHR to include capabilities beyond merely hospital data is important. Achieving this requires an open and honest discussion about the role of the PHR, potential obstacles and how to coordinate efforts among different stakeholders.

## 1. Introduction

A Patient Portal (PP), also known as a Personal Health Record (PHR), is a platform managed by patients that contains their health information. This includes their medical record number, name, date of birth, blood group, eligibility information, referral, hospital, emergency contacts and other details. The portal allows patients to access laboratory and radiology results, medication information and other reports. They can also request certain services from their healthcare provider and use the portal as a communication channel [1,2].

Patient portals can be tethered to the provider system, be stand-alone or in a hybrid form that integrates with the provider’s system [3,4]. In one study that assessed the adoption of a PHR in a tertiary hospital, 70% of patients were interested in using the PHR. However, the authors indicated that a more objective approach was needed to confirm the self-reported results [5]. Similarly, a study in Korea found that more than 60% of patients were using a PHR [6].

Although PHR adoption was estimated to exceed 75% by 2020 in the USA, the primary factor limiting the broader utilization of PHR functionality lies in the capacity of healthcare providers to help and deploy such functionalities for the sake of patients [7]. A Personal Health Record (PHR) can improve health outcomes and provide more accurate data in hospital Electronic Medical Record (EMR) systems [8]. However, these positive benefits can only be achieved if patients are able to easily utilize the PHR and comprehend the data within it. Providers must also ensure that they provide accurate data, address legal concerns and promptly respond to patient requests [9]. Moreover, a systematic review assessed the factors hindering the wider adoption of PHRs from the patient side and found that perceived usefulness, privacy and security concerns and internet access were the main barriers [10]. Another study revealed that performance expectancy, effort expectancy, habit and self-perception were the main drivers of patients’ adoption of the PHR [11].

Generally, most studies identify adoption barriers related to low socioeconomic status, and most recommendations are presented to elevate patient literacy from the health and technology sides [12,13]. The study connects patients’ multiple comorbidities and willingness to use a PHR. The study discloses that patients with multiple chronic conditions (MCC) use a PHR three times more than patients with no chronic disease [14]. Wells and his colleagues have suggested organizational strategies to improve the adoption of PHRs. These strategies include organizational efforts such as establishing a clear vision, governance, process redesign, providing training, IT support, monitoring and incentives. Other strategies are aimed at improving patient intake through professional encouragement [15].

Recently, the process of creating a complete Patient Health Record (PHR) has presented even more challenges. One of these is that the information in the record is only partially obtained from hospital Electronic Medical Record (EMRs), with some data being omitted. Additionally, there is a need to incorporate data from other sources, such as wearable technology and patient-generated data, into the PHR [16,17].

The above-provided literature suggests insufficient research on the PHR. Most studies only concentrate on patient adoption or attitudes toward hospital-provided PHRs in one organization. Few studies examine the essential functions of a PHR or its overall effect on patient outcomes. Therefore, the objectives of this study were to answer questions related to how patients perceive the PHR in multiple tertiary hospitals, and the preferred functions, challenges and the overall impact of PHRs on their healthcare outcomes.

## 2. Materials and Methods

This study was a descriptive cross-sectional survey that recruited participants from four tertiary hospitals in Riyadh City. Each hospital aimed to provide a sample size of 100, resulting in a total of 400 participants for the study. To ensure the recruitment of the required number of patients, we invited 120 patients from each hospital. The objectives were to measure the patients’ attitudes toward the hospital’s Personal Health Record (PHR) using the Technology Acceptance Model (TAM). In TAM, the intention of individuals to use certain technology (PHR in our study) is determined by their attitudes. A positive attitude is formed through perceiving that the PHR will be useful (Perceived Usefulness, PU: enhance the task performance) or perceiving that the PHR will be easy to use (Perceived Ease of Use, PEOU: free of physical and mental effort). The final construct is the influence of social norms, which reflect an individual’s perceptions regarding how others would expect him/her to behave. To determine the preferred functions of the PHR, a list of all functions was provided, including the following functions: view my medical record, schedule an appointment, refill medication, monitor patient data and receive targeted education. The selected functions were abstracted from the literature, and patients in our study were asked to select the needed function and could add more. To evaluate the impact of the PHR, three outcomes were assessed: monitoring health status, improving patient–doctor interaction and enhancing the quality of care. The survey had two questions about the main challenges (concerns about privacy and accuracy of information).

The survey was reviewed by experts, and a pilot study was conducted to confirm its validity and reliability. The test results showed that the questions effectively distinguished between different concepts. Additionally, the reliability of the survey was assessed, and the reliability of the three constructs, “perceived PHR as useful”, “perceived PHR as easy to use” and “influenced by social norms”, were tested through a reliability test. The resulting Cronbach’s alpha values were 0.811, 0.836, and 0.822. (i.e., alpha = 0.70 or above).

Furthermore, the results were analyzed through descriptive means, as frequencies and percentages, to record and interpret patients’ attitudes toward the PHR, the required functions, the impact of the PHR on patients’ outcomes and the challenges of PHR adoption. Moreover, a chi-square test, one-way ANOVA and Kruskal–Wallis test of independence were used to determine whether there were any statistically significant differences in patients’ responses based on their characteristics and perceptions. A *p*-value of 0.05 or less was chosen to be the cut-off point to consider a difference statistically significant.

Prior to conducting the study, we obtained ethical approval from the King Abdullah International Medical Research Center (KAIMRC) with approval number SP22R/191/07 and ensured that patient confidentiality would be maintained. We obtained informed consent from participants and assured them that only aggregated data would be reported. The study occurred from September 2022 to February 2023 and included the main tertiary hospitals in Riyadh City, Saudi Arabia.

## 3. Results

We received 396 completed surveys from the four tertiary hospitals out of the 480 invitations sent, resulting in a response rate of 82.5%. The response rate improved after sending two reminders, one and two weeks after the original invitation. The general characteristics of the sample indicated that 268 of the participants (68%) were males. Most respondents (83%) were between 25 and 44 years old. Most participants held a diploma or higher (91%). Furthermore, most of the participants did not have any chronic disease (81%), 14% had one chronic disease and 5% had two or more chronic diseases. For a detailed overview of the demographic characteristics of the participants, Table 1 presents the findings across the four hospitals examined.

To assess the participants’ attitudes toward using EPP, we found that 93% of the participants intended to use the PHR to interact with their physicians, 94% perceived the PHR as a useful technology and 94% perceived the PHR as easy to use. Additionally, we found that 94% would use the PHR if their friends or family members were using it, indicating that social norms played a role in their decision making. See Table 2.

For the required functions preferred by the participants, Table 3 demonstrates the functions likely to be used by the participants. Among the many listed functions, most respondents showed a high level of consensus regarding the need to view medical records (77%), book or reschedule appointments (88%), refill medication (90%), monitor patient data, such as daily weight/blood glucose/physical exercise activity (70%), and receive targeted education from physicians enabling disease self-management (78%).

According to the study, the effectiveness of the PHR for patients’ outcomes was evaluated based on several measures. These measures included the ability of the PHR to monitor the health status, track health indicators, promote a healthier lifestyle, provide support during critical times of sickness and save time when using the PHR. The results showed that 54% of participants perceived a positive impact of the PHR on monitoring their health status. However, only half of the participants believed that the PHR would improve patient–doctor interactions, and less than half of them thought that it could enhance the quality of care. The impacts of the PHR on each outcome and its items are described in Table 4.

According to the findings, a significant number of individuals, approximately 209 (54%), appeared to be apprehensive about the privacy of their PHR when utilizing these services. Conversely, only 182 (46%) of participants reported being concerned about the accuracy of their information, indicating that privacy concerns were a more pressing issue for PHR users.

On the other hand, we examined the relationships between participants demographic characteristics (sex, age, qualification and comorbidity) and their intention to use the PHR through using Pearson chi-square tests, as illustrated in Table 5. The results revealed that the intention to use the PHR was not statistically significantly associated with these demographic factors (i.e., *p*-values 0.64, 0.73, 0.95, 0.64, respectively).

Furthermore, one-way ANOVA and Kruskal–Wallis tests were used to assess whether there were statistically significant differences in responses on the attitudes toward PHR based on the sample hospital; we can conclude that the four hospital samples did not statistically differ in terms of the mean attitude scores regarding the use of EPP (i.e., *p*-values were 0.53, 0.076, 0.322). Table 6 describes the participants’ attitudes toward using the PHR.

## 4. Discussion

The study aimed to assess the essential elements of Personal Health Records (PHRs). We discovered that PHR adoption is not a significant issue based on our research and the literature. Most hospitals offer a tethered PHR, which is a basic extension of their Electronic Medical Record (EMR) that provides access to basic functions. For the first research question, our study found that over 90% of patients strongly intended to use Personal Health Records (PHRs), and this indicates a high adoption rate. One study reported that 70% of patients currently used a PHR, while another showed that 75% would use a PHR if available [18,19]. This indicates that there is no major issue that hinders the use of a PHR if it is made available to patients. Furthermore, our findings indicate that the sociodemographic characteristics of patients do not correlate with their intention to use the PHR. Another study confirmed our findings that patients’ intention to use PHR was not associated with gender, age or the presence of comorbidities [20]. In contrast, many other studies found that age, race, literacy, annual income and the presence of comorbidities were associated with the greater adoption of PHRs [14,21,22].

In the second part of the study, addressing the required functionalities of the PHR, more than 70% of our sample indicated that they wished to view their medical records, request appointments and medications, monitor their health data and receive customized educational materials. Similarly, a recent systematic review indicated that patients would need a PHR to communicate with healthcare providers, manage medication and schedule appointments [23].

Our most interesting findings are related to the impact of current PHRs; less than half of our sample claimed that the PHRs did not help them effectively in monitoring their health status, improving patient–doctor interaction and enhancing the quality of care. This should be the focus across all healthcare organizations. The current concept and scope of the PHR should be changed, not only regarding the functionality. This can be attributed to the poor integration of patients’ main information components in EMR and EHR across different providers [24]. The PHR should enable patients to manage their health information holistically in a manner that helps them to compile all hospital information with their generated information.

Technological advancements enable consumers to collect data in and outside of hospital, whether sick or simply performing their normal daily activities. The current technology, including but not limited to the Internet of Things, wearable devices, mobile applications and monitoring devices, emphasizes a different and evolving concept of PHR. Nevertheless, most hospitals struggle with integrating and using patient-generated data in their systems for many reasons. Many studies indicate that a lack of resources, the quality of patient-generated data and preferences for data use are among the main barriers to the integration of patients’ external and EHR data [25]. To improve integration, it is important to focus on targeted solutions. Hospitals must update their information infrastructures and optimize how they capture and compile patient-generated data in their systems. Furthermore, current EHR systems should be more extensible to encompass such external sources. Artificial intelligence can offer the ability to conduct spontaneous data checks, which can resolve concerns about the accuracy of data received from external systems and patients. Furthermore, liability, accountability and the issue of reimbursement for such activities should be discussed openly with all stakeholders. The next-generation healthcare system should prioritize providing value to patients, focusing on population health management. This holistic approach is expected to emerge in the near future [26].

Additionally, hospitals should focus on automating more care processes, encouraging healthcare providers and educating them on the value of capturing data about patients, not only related to the current episodes of care. As hospitals are required to achieve cost savings and receive incentives to maintain patients’ health, they may need to introduce incentives to care providers to provide integrated and coordinated care. Furthermore, hospitals are obligated to elevate patients’ health literacy. Hence, patients should undergo educational sessions regarding the capabilities of the available information technology solutions, including EMR, PHR, mobile applications and current emerging technologies [15]. In addition to this, professional encouragement, using social media and even traditional media, should be designed to optimize patients’ capabilities to manage not only their health data but, more importantly, their health status. The new PHR should move from simply a record with little selective information from the hospital to a tool that aims to enhance health, from the notion of scattered records to an outcome-oriented tool. There is little value in providing a patient with lab results, for example, if he/she cannot use them in terms of maintaining or enhancing his/her overall health. Furthermore, personal records should utilize the power of pervasive technology; data are everywhere, and even a step count, for example, is important for healthcare providers to infer information about the patient’s lifestyle. Calory tracking can trigger proactive care, as this can indicate an escalating risk for cardiovascular or metabolic diseases.

On the other hand, it is acknowledged that there is a digital divide in the use of information technology, and this can be attributed to many factors [21]. However, an unspoken issue is the disparity and different maturity levels observed in healthcare organizations adopting information technology solutions [27]. With government support and more engaged healthcare providers, this could be resolved to enable smooth and integrated care across all healthcare providers, including the most important player, the patient. This reflects the vital role of the primary care provider as a liaison to work to harmonize the provided care services and oversee and maintain their population’s health [28,29].

In conclusion, PHR systems are being widely adopted by patients regardless of their providers. However, the utility of the PHR system, through impacting patient health, has many deficiencies. Our study provides a snapshot of how PHR systems are currently being used. However, we did not assess the reasons behind the suboptimal impact of PHRs. Although the study assessed patients’ attitudes toward PHRs in four hospitals, generalizing the findings to another context should be done cautiously. As this was a descriptive study, it was naturally prone to bias related to self-presentation and could not determine causality between the study constructs and outcomes. Further studies should examine these shortcomings in greater depth by investigating the poor impact of the PHR on patients’ outcomes, utilizing different study designs, qualitative and quantitative, with data collected from all stakeholders.

## 5. Conclusions

The Personal Health Record is an indispensable tool in optimizing an individual’s health. The status of the adoption of PHRs indicates optimal diffusion, as indicated by our study. Additionally, the current PHRs provide essential functions; however, we failed to draw a connection between these functions and better outcomes for patient health. With emerging technologies, more data are being generated and must be incorporated into PHRs to provide an accurate and complete picture of patient health. The scope of the PHR must evolve to include capabilities beyond hospital data. However, hospitals must excel in their data capturing, compilation and dissemination by updating their current technical and administrative processes to expedite the transition toward a more meaningful and effective PHR. Nevertheless, hospitals’ roles should not end with their in-house data; they should encourage and empower patients to exploit all available technologies to bring together internal and external data and clinical and non-clinical data, to enrich the patient experience and support customized, integrated and coordinated care. This is not attainable without first initiating an open and honest discussion about the desired roles of the PHR, the obstacles and strategies to harmonize efforts between all stakeholders, regulatory bodies, care providers, funders and patient representatives.

## Figures and Tables

**Table 1:** Demographic characteristics of the respondents.

Characteristic		H1	H2	H3	H4	Overall
		n (%)	n (%)	n (%)	n (%)	n (%)
Frequency (n), %		95 (24%)	100 (25.25%)	99 (25%)	102 (25.75%)	396 (100%)
Sex	Male	78 (82)	62 (62)	59 (60)	69 (68)	268 (67.7)
	Female	17 (18)	38 (38)	40 (40)	33 (32)	128 (32.3)
Age	18–24	9 (9)	7 (7)	8 (8.1)	4 (4)	28 (7)
	25–34	49 (52)	47 (47)	43 (43.4)	44 (43)	183 (46.2)
	35–44	27 (28)	36 (36)	36 (36.4)	47 (46)	146 (36.9)
	45–55	10 (11)	8 (8)	10 (10.1)	6 (6)	34 (8.6)
	50+	0 (0)	2 (2)	2 (2)	1 (1)	5 (1.3)
Qualification	High school	10 (11)	7 (7)	10 (10)	9 (8.8)	36 (9)
	Diploma	11 (12)	28 (28)	14 (14)	22 (21.6)	75 (19)
	University	74 (78)	65 (65)	75 (76)	71 (69.6)	285 (72)
Comorbidity	None	82 (86)	77 (77)	83 (84)	79 (77.4)	321 (81.1)
	One disease	13 (14)	15 (15)	10 (10)	16 (15.7)	54 (13.6)
	Two diseases	0 (0)	4 (4)	4 (4)	6 (5.9)	14 (3.5)
	More than two	0 (0)	4 (4)	2 (2)	1 (1)	7 (1.8)

**Table 2:** Assessment of patients’ attitudes toward PHR across the four tertiary hospitals.

Acceptance Constructs	Hospital 1	Hospital 2	Hospital 3	Hospital 4	Overall
	n (%)	n (%)	n (%)	n (%)	n (%)
Intend to use the PHR. No Yes					
	11 (12)	7 (7)	6 (6)	4(4)	28 (7)
	84 (88)	93 (92)	93 (94)	98 (96)	368 (93)
2. Perceived PHR as useful technology No Yes					
	12 (13)	7 (7)	2 (2)	2 (2)	23 (5.8)
	83 (87)	93 (93)	97 (98)	100 (98)	373 (94.2)
3. Perceived PHR as easy to use. No Yes					
	10 (11)	4 (4)	6 (6)	6 (6)	26 (6.6)
	85 (89)	96 (96)	93 (94)	96 (94)	370 (93.4)
4. Influenced by social norms. No Yes					
	12 (13)	6 (6)	4 (4)	2 (2)	24 (6)
	83 (87)	94 (94)	95 (96)	100 (98)	372 (94)

**Table 3:** PHR required functions preferred by the participants.

Functions	H1	H2	H3	H4	Overall
	n (%)	n (%)	n (%)	n (%)	n (%)
View my medical record					
Agree/Strongly agree	70 (74)	72 (73)	86 (87)	76 (75)	304 (77)
Schedule an appointment					
Agree/Strongly agree	84 (88)	87 (87)	91 (92)	86 (84)	348 (88)
Refill medication					
Agree/Strongly agree	84 (88)	87 (87)	97 (98)	90 (88)	358 (90)
Monitor patient data					
Agree/Strongly agree	63 (66)	64 (64)	75 (76)	75 (74)	277 (70)
Receive targeted education					
Agree/Strongly agree	79 (83)	75 (75)	79 (80)	76 (75)	309 (78)

**Table 4:** Impact of PHR on patient outcomes.

Impact of PHR on Patient Outcomes		n (%)	Overall (%)
Monitor health status	Track health indicators	210 (53)	54
	Easier to have a healthier life	208 (52.5)	
	Support during a critical time of sickness	183 (46)	
	Using PHR saves time	253 (64)	
Improve patient–doctor interaction	Communicate with physicians	199 (50)	50
	Easy to post information	179 (45)	
	Easy to follow information	214 (54)	
Enhance the quality of care	PHR gives greater control over diseases	214 (54)	47
	PHR enhances the quality of care	159 (40)	

**Table 5:** Chi-square tests across participants’ characteristics and intention to use PHR.

Characteristic		Not Intend to Use PHR (%)	Intend to Use PHR (%)	All Respondents (%)	Chi-Square *p*-Value
Gender	Male	73.3	67.6	68	0.644
	Female	26.7	32.4	32	
Age	18–24	0	7.4	7.4	0.732
	25–34	53.3	46.3	46.8	
	35–44	33.3	36.7	36.5	
	45–55	13.3	8	8.4	
	50+	0	1.1	1	
Qualification	High school	6.7	9	8.9	0.952
	Diploma	20	19.1	19.2	
	University	73.3	71.8	71.9	
Comorbidity	None	93.3	80.3	81.3	0.641
	One disease	6.7	14.4	13.8	
	Two diseases	0	3.7	3.4	
	More than two	0	1.6	1.5	

Chi-square test of independence. A *p*-value < 0.05 was considered statistically significant.

**Table 6:** Participants’ attitudes regarding the use of PHR, median and IQR according to hospital.

Construct		Mean, Median, IQR	One-Way ANOVA (*p*-Value)	Kruskal–Wallis Test (*p*-Value)
PHR service features likely to be used			0.513	0.533
	HI	4.17, 4.40, 1.25		
	H2	4.02, 4.20, 1.00		
	H3	4.26, 4.40, 0.80		
	H4	4.14, 4.30, 1.40		
PHR usefulness			0.107	0.076
	HI	3.72, 4.00, 1.88		
	H2	3.72, 3.42, 2.00		
	H3	3.33, 3.38, 1.25		
	H4	3.33, 3.50, 2.25		
PHR ease of use			0.361	0.322
	HI	3.30, 3.63, 1.94		
	H2	3.58, 4.00, 2.00		
	H3	3.19, 3.00, 1.56		
	H4	3.33, 3.38, 2.38		

Integrate the title with the one presented in the table.

## Data Availability

Not applicable.
